# Association mapping of common bacterial blight resistance QTL in Ontario bean breeding populations

**DOI:** 10.1186/1471-2229-11-52

**Published:** 2011-03-24

**Authors:** Chun Shi, Alireza Navabi, Kangfu Yu

**Affiliations:** 1 Agriculture and Agri-Food Canada, Greenhouse and Processing Crops Research Centre, Harrow, ON, N0R 1G0, Canada; 2 Department of Plant Agriculture, Crop Science Building, University of Guelph, Guelph, ON, N1G 2W1, Canada

## Abstract

**Background:**

Common bacterial blight (CBB), incited by *Xanthomonas axonopodis *pv. *phaseoli *(*Xap*), is a major yield-limiting factor of common bean (*Phaseolus vulgaris *L.) production around the world. Host resistance is practically the most effective and environmentally-sound approach to control CBB. Unlike conventional QTL discovery strategies, in which bi-parental populations (F_2_, RIL, or DH) need to be developed, association mapping-based strategies can use plant breeding populations to synchronize QTL discovery and cultivar development.

**Results:**

A population of 469 dry bean lines of different market classes representing plant materials routinely developed in a bean breeding program were used. Of them, 395 lines were evaluated for CBB resistance at 14 and 21 DAI (Days After Inoculation) in the summer of 2009 in an artificially inoculated CBB nursery in south-western Ontario. All lines were genotyped using 132 SNPs (Single Nucleotide Polymorphisms) evenly distributed across the genome. Of the 132 SNPs, 26 SNPs had more than 20% missing data, 12 SNPs were monomorphic, and 17 SNPs had a MAF (Minor Allelic Frequency) of less than 0.20, therefore only 75 SNPs were used for association study, based on one SNP per locus. The best possible population structure was to assign 36% and 64% of the lines into Andean and Mesoamerican subgroups, respectively. Kinship analysis also revealed complex familial relationships among all lines, which corresponds with the known pedigree history. MLM (Mixed Linear Model) analysis, including population structure and kinship, was used to discover marker-trait associations. Eighteen and 22 markers were significantly associated with CBB rating at 14 and 21 DAI, respectively. Fourteen markers were significant for both dates and the markers UBC420, SU91, g321, g471, and g796 were highly significant (p ≤ 0.001). Furthermore, 12 significant SNP markers were co-localized with or close to the CBB-QTLs identified previously in bi-parental QTL mapping studies.

**Conclusions:**

This study demonstrated that association mapping using a reasonable number of markers, distributed across the genome and with application of plant materials that are routinely developed in a plant breeding program can detect significant QTLs for traits of interest.

## Background

Common bean (*Phaseolus vulgaris *L.) is a diploid (2n = 2x = 22) annual species, and is predominantly self-pollinating [1]. It is the most important grain legume for direct human consumption. Its nutritional composition includes complex carbohydrates (e.g. fibre, resistant starch, and oligosaccharides), vegetable protein, important vitamins and minerals like folate and iron as well as antioxidants and only very small amounts of fat [1]. In 2006, the bean industry was valued at $1.2 billion and $180 million in USA and Canada, respectively (http://www.pulsecanada.com/).

Common bacterial blight (CBB), incited by *Xanthomonas axonopodis *pv. *phaseoli *(*Xap*), is a serious seed-borne disease in both temperate and tropical bean production zones [2]. Yield losses can exceed 40% [2]. Control measures for CBB include the use of disease-free seed, crop rotation, application of copper-based products and antibiotics, and cultivation of resistant varieties [2]. In practice, host resistance is the most effective and environmentally-sound approach to control CBB [2]. Over the years, bean breeders have utilized different sources of resistance from *P. vulgaris *and its close relatives in intra- and inter-specific crosses to improve CBB resistance in beans. These sources include the common bean cultivar Montana No. 5 and introduction line PI207262, tepary bean (*Phaseolus acutifolius *L.) introduction lines (PI319443 and PI440795), and scarlet runner bean (*Phaseolus coccineus *L.) [3]. In Canada, tepary bean introduction lines PI319443 and PI440795 have provided the major sources of resistance to CBB in different bean breeding programs. The germplasm lines HR45 [4] and HR67 [5] and the elite line HR199-4857 have obtained their resistance through crosses to XAN159, which was developed through interspecific crosses to PI319443 [6]. The cultivar OAC-Rex [7], on the other hand, was developed through crosses to a breeding line, which was derived from interspecific crosses to PI440795. More recently, the elite line, OAC 07-2 (Smith et al. unpublished), was developed through crosses of OAC-Rex to cultivar Kippen, which is derived from crosses involving HR45. Previous studies have reported molecular markers tightly linked to CBB resistance QTLs in both HR45 [8,9] and OAC-Rex [10]. The two SCAR (Sequenced Characterized Amplified Region) markers, SU91 and UBC420, have been of particular interest to bean breeding programs for marker-aided selection for CBB resistance [11].

Traditionally, QTL mapping approaches have been based on the analysis of populations derived from bi-parental crosses that segregated for trait(s) of interest. To date, at least 24 different CBB resistance QTLs have been reported across all eleven linkage groups of common bean [3]. However, these QTLs were mapped in eight different bi-parental populations and poorly co-localize [3], thus markers linked to these QTLs are not immediately available for use in bean breeding. QTL effects are required to be validated in other genetic backgrounds prior to widespread application of QTL-linked markers in marker-assisted selection (MAS). Alternatively, association mapping is a new QTL mapping approach that can use natural populations, the collection of cultivars released over years, and the material within a breeding program [12]. These types of populations, or a subset of these may represent a smaller set of the available genetic diversity within a breeding program. Collections of these lines may provide great potential for applied association mapping experiments because they are routinely evaluated in the breeding programs and regional trials to assess their local adaptation or response to biotic and/or abiotic stresses [12].

Association mapping is increasingly being utilized to detect marker-QTL linkage associations using plant materials routinely developed in breeding programs. Compared with conventional QTL mapping approaches, association mapping using breeding populations may be a more practical approach for cultivar development, considering that markers linked to major QTL can immediately be utilized in MAS, once new QTLs are identified. For instance, in soybean (*Glycine max *L. Merr.) two markers, Satt114 and Satt239, were found to be associated with iron deficiency chlorosis loci using advance breeding lines [13]. In rice (*Oryza sativa *L.), microsatellite markers associated with yield and its components were identified in a variety trial, and many of them were located in regions where QTL had previously been identified [14]. Association mapping studies have also been used to investigate the genetic diversity within crop species. High levels of LD (Linkage Disequilibrium) (pairwise LD: 56%; average *r^2 ^*= 0.1) was found in common bean [15]. Much higher LD was observed in domesticated populations (pairwise LD: 57.3%; average *r^2 ^*= 0.18) compared to wild populations (pairwise LD: 31.5%; average *r^2 ^*= 0.08) [15]. In the presence of high LD, lower marker density is required for a target region with greater potential for detecting markers strongly associated with the target gene polymorphism, even if distant physically. Thus, whole-genome-scan association study is feasible for bean domestic populations [15].

In association mapping, where unlike conventional QTL mapping, populations of un-structurally related individuals are employed, it is important to consider population structure and kinship among individuals, because false associations may be detected due to the confounding effects of population admixture [12]. This may indeed be the case for populations drawn from large collections, breeding materials, or from released cultivars. Therefore, it is important to apply appropriate statistical methods that account for population structure and kinship among individuals. A Mixed Linear Model (MLM) approach has been developed to account for multiple levels of relatedness simultaneously as determined by kinship estimates based on a set of random genetic markers [16]. This model has been proven useful in genome-wide association studies to control the biased that may be caused by population structure and relatedness in other species e.g., maize (*Zea mays *L.) [16], rice [14]. Another issue for association mapping is reliability, an issue of particular concern when the goal is to discover marker/trait associations that have broad application [16].

Single Nucleotide Polymorphic (SNP) markers are currently known as valuable markers for genotyping because of their abundance, stability, and simplicity. The total number of SNPs in cultivated bean is estimated to be in the range of 3-4 millions, based on the rate of 237 SNPs observed in 38.2 kbp of sequence in 6 diverse genotypes [17]. So far, five methods have been used for SNP genotyping in common bean. CAPS (Cleaved Amplified Polymorphic Sequences) and dCAPS (derived Cleaved Amplified Polymorphic Sequences) techniques have been used to convert EST based polymorphisms into SNP markers [18]. Another approach is a high-throughput system named Luminex-100 (http://www.luminexcorp.com) which was used to confirm SNP calls in DNA from 10 common bean genotypes, finding 2.5% of SNPs were miscalled and 1% had no signal as compared with direct sequencing [19]. In an effort to simplify SNP analysis, Galeano et al. [20] used *CEL I *mismatch digestions to analyze and map SNP-based, EST-derived markers, finding that the method worked well with SNPs located in the middle of amplification fragments and that digestion products could be visualized on agarose gels. Single strand conformation polymorphism (SSCP) technology was employed to develop and map EST based markers, which resulted in identification of a total of 118 new marker loci in DOR364 × G19833 mapping population [21]. Latest attempt was to validate predicted SNPs using 1,050-plex GoldenGate assay from Illumina (http://www.illumina.com). 79% (827 of 1,050) SNPs produced a working GoldenGate assay [22]. Another high-throughput system, named Sequenom iPLEX Gold genotyping technology provides an ideal technique for medium sized projects, when scoring between 5 and 400 SNP markers on hundreds to a few thousands of DNA samples [23]. A major advantage with this technology is that it is highly flexible, since there are no SNP type restrictions for the construction of the panel [23]. The Sequenom platform has been used successfully in a wide range of plant genotyping applications, for instance, SNP validation in sugarcane (*Poaceae Saccharum *L.) [24], high-throughput genotyping in rice [25] and wheat (*Triticum *spp.) [26], and variety identification in barley (*Hordeum vulgare *L.) [27].

The objectives of our study were to 1) apply unified MLM association mapping approach to identify CBB resistance loci in Ontario bean breeding materials and 2) evaluate whether association mapping can be used effectively to discover CBB resistance QTLs using SNP genotyping of plant materials, routinely developed in a bean breeding program.

## Results

### 1. Phenotypic analysis of CBB resistance

**Figure 1:**
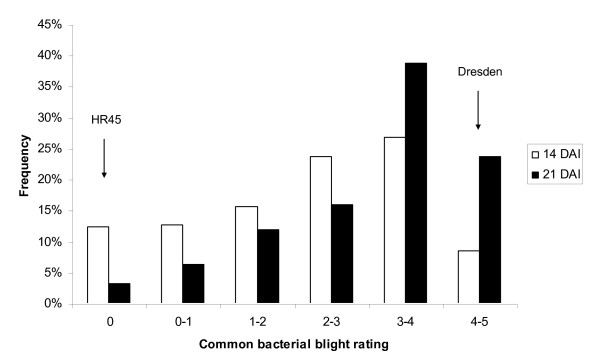
**The frequency distribution of CBB ratings of the entries in 2009 CBB nursery**. Rating scales: 0 = no symptoms and 5 = more than 80% of inoculated areas showing symptoms.

CBB resistance in common bean is a complex trait, known to be controlled by both major and minor genetic factors [3]. Each line was rated twice for CBB resistance. Resistant check HR45 was scored 0 at both disease observation dates, whereas susceptible check Dresden was scored 5 (Figure 1). The frequency distribution of CBB severity scores showed a continuous variation with population mean shifted towards susceptibility (Figure 1). The Kolmogorov-Smirnov test of normality for the whole population was significant (P ≤ 0.05) for both 14 and 21 DAI.

### 2. Summary of SNP performance and quality

Over 99% of data points were identically scored in the 14 repeated samples evenly distributed over all 96-well plates. Only one type of genotyping error was found in three SNP assays, where a SNP was called in one plate but uncalled in the repeated sample in another plate. Thus, the reproducibility and reliability of SNP assay were high and comparable with other SNP assays in plant species.

Of the 132 bean SNPs used in the SNP assay, 106 SNPs (80.3%) were successfully called in the 469 lines with less than 20% missing data points. Of them, 12 SNPs were monomorphic in all 469 lines and 94 SNPs were polymorphic. The 94 polymorphic SNPs were retained for the next stage of screening.

Bean cultivars, and advanced breeding lines are generally homozygous and highly homogeneous. However, complete homozygosity is practically unattainable, and slight levels of heterogeneity may be present for small number of loci. In the present study, the number of genotypes heterogeneous for the two alleles at a SNP locus ranged from 1 to 300 at 69 of the 94 SNP loci. Although heterozygosity ranged from 0 to 0.62 with one SNP having diversity values in excess of 0.3, the heterozygosity average was 0.02, well within the expected ranges for residual heterozygosity found in bean cultivars.

All SNPs were well distributed across the 11 bean chromosomes with a genome coverage ranging from 6 SNPs on chromosome 6 to 11 SNPs on chromosome 10 (Table 1). This represented 85 loci with an average of 1.1 SNPs per locus. Among the 85 loci, 76 contained only one SNP, and the other 9 contained 2 SNPs per locus (Table 1). Based on the observation of the 469 lines, Minor Allelic Frequency (MAF) of the 94 SNPs varied from 0.01 to 0.49 with an average frequency of 0.31. Of the 94 SNPs, 77 had a MAF value greater than 0.20 (Table 1). In order to extract the most useful information from the SNP data, a total of 75 SNPs were selected for further data analysis (Additional file 1). The selection criterion is only one SNP per locus with a MAF value greater than 0.2.

**Table 1:** Summary of SNPs used in this study

**Chr**.	SNP Number	Unique loci		Minor Allelic Frequency	
		
		2 SNPs	1 SNP	≤0.2	>0.2
1	10	1	8	1	9
2	8	2	4	2	6
3	8		8	1	7
4	7		7		7
5	9	2	5	3	6
6	6		6		6
7	10	1	8	3	7
8	9	1	7	2	7
9	9	1	7	1	8
10	11	1	9	3	8
11	7		7	1	6
Total	94	9	76	17	77

### 3. Population Structure

**Figure 2:**
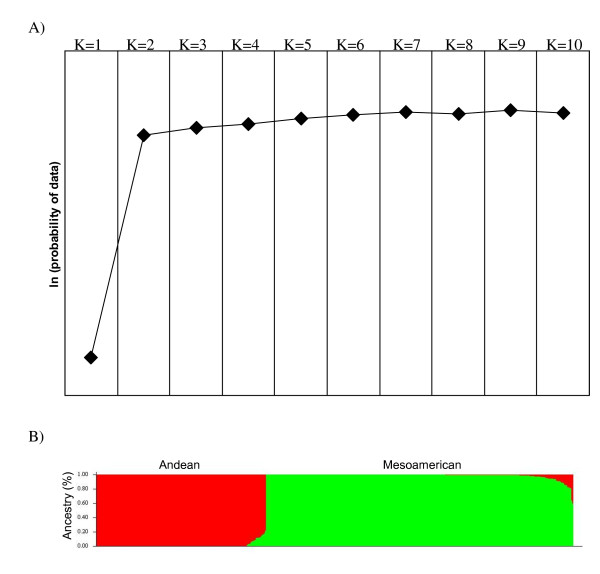
**Population structure estimation**. A) Estimated ln (probability of the data), which was calculated for K ranges from 1 to 10; B) Estimated population structure at K = 2. Each individual is represented by a thin vertical line, which is partitioned into 2 coloured segments that represent the individual membership to the 2 clusters.

The software STUCTURE was run for K (number of fixed subgroups or clusters) ranging from 1 to 10 on the entire set of breeding lines using all SNPs scored as biallelic markers. The likelihood value of this analysis is shown in Figure 2. Likelihood increases continuously and no obvious inflection point were observed. This could imply that the lines included in the analysis were very diverse. However, the most significant change was observed when K was increased from one to two, which corresponds with the origin, pedigree, and breeding history of the breeding populations that can be divided as either Mesoamerican or Andean subgroups. Therefore, the Structure results of K = 2 was considered the best possible partition as they showed a high consistency with known pedigree history and geographic/gene pool origin of the material (Figure 2A). Thus, 36% (169 of 469) of the lines were assigned to Andean subgroup, whereas 64% (300 of 469) of the lines to the Mesoamerican subgroup. A further study of the partitioning of lines can be seen in Figure 2B, which is the graphical representation of the placement of each line in the study into its corresponding cluster, for K = 2. Such a graph shows the number of lines in each cluster, and the percent mixing of each line within each cluster, a useful visualization of admixture.

### 4. Relative Kinship

**Figure 3:**
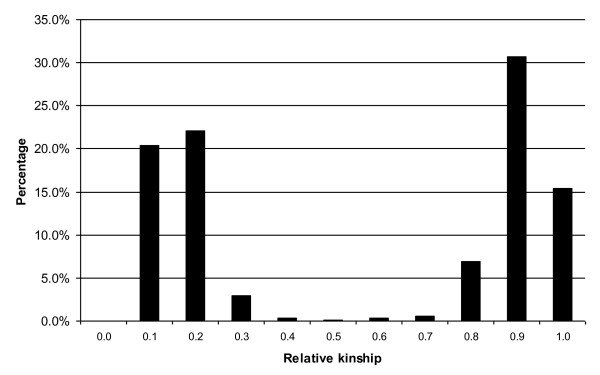
Frequency distribution of pairwise relative kinship values

Molecular markers can be used to estimate the relative kinship between pairs of individuals in a study, which provides useful information for quantitative inheritance studies. The relative kinship reflects the approximate identity between two given individuals over the average probability of identity between two random individuals [28]. In this study, 75 informative SNPs with MAF>0.2 and little or no missing data were used to estimate the relative kinship in the set of 469 lines. As shown in Figure 3, about 42.5% of the pairwise kinship estimates were from 0 to 0.2, indicating that the lines were distantly related or unrelated. Meanwhile, 53.1% of the pairwise kinship estimates were from 0.8 to 1, indicating that the lines were closely related. Therefore, the kinship analysis indicates complex familial relationships among the 469 lines, matching with the known pedigree history mentioned in Table 2.

**Table 2:** List of 469 genotypes used for molecular marker analysis

*a) Lines selected from the Ancestry of Navy Bean Varieties Registered in Canada Since 1930*							
AC Hensall	AC Mariner	AC Mast	AC Skipper	AC Trident	Centralia	Clipper	Corvette
CRAN74	Crestwood	Domino	Dresden	Envoy	Fleetside	Fleetwood	Galley
Harofleet	Harokent	Harowood	HR13	HR14 818	HR199 4857	HR45	HR67
ICA Pijao	Kippen	M03	Magdalena	Michelite	Midland	Midnight	Mitchell
Nautica	Navigator	Nep-2	Northland	NY5268	NZ Upright	OAC 07-2	OAC Cygnus
OAC Gryphon	OAC Laser	OAC Rex	OAC Rico	OAC Silvercreek	OAC Speedvale	OAC Sprint	OAC Thunder
PI440795	Redhawk	ROCKET	Sacramento LRK	Saginaw	Sanliac	Seafarer	Shetland
Stinger	Swan Valley	Targ	Vista	Wesland	XAN159		

***b) Other lines and cultivars***							

2793CBB	A300	AB	AC Elk	AC Pintoba	AC Redbond	AC Compass	BAT93
Calmont	Cruiser	Dublin	Etna	G122	Harohawk	Hooter	HR200
JaloEEP558	Lightning	Lyrik	MBE7	Montcalm	N203	NY2114-12	Othello
RCX6067	RCX6079	RedRider	OAC RedStar	T9905			

***c) Advanced breeding lines in the AAFC/University of Guelph Bean Breeding Program***							

***Advance yield trial (n = 116)***							

1^st ^Group: coloured bean (CB AYT)				2^nd ^Group: white bean, early maturity (WB AYT E)			
3^rd ^Group: white bean, medium maturity (WB AYT M)				4^th ^Group: white bean, later maturity (WB AYT L)			

***Preliminary yield trial (n = 262)***							

1^st ^Group: coloured bean, early maturity (CB PYT E)				2^nd ^Group: coloured bean, medium maturity (CB PYT M)			
3^rd ^Group: coloured bean, later maturity (CB PYT L)				4^th ^Group: white bean, early maturity (WB PYT E)			
5^th ^Group: white bean, medium maturity (WB PYT M)				6^th ^Group: white bean, later maturity (WB PYT L)			

### 5. Association mapping

**Figure 4:**
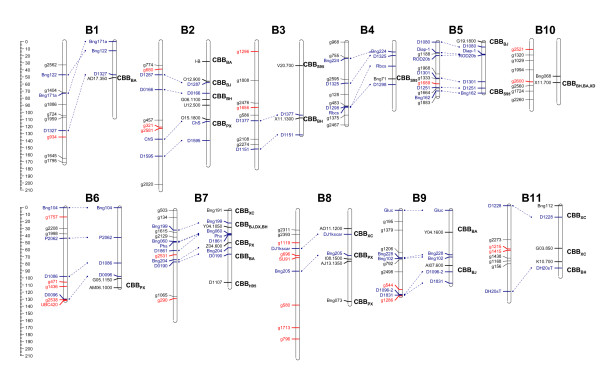
**The distribution of molecular markers co-localized with previously indentified QTLs associated to CBB resistance**. For each linkage group, the map on the left is reproduced from McClean(2007) map (http://www.comparative-legumes.org/) [33], the map on the right is reproduced from comprehensive Freyre (1998) map (http://www.comparative-legumes.org/), adopted from Miklas et al (2006) [3]. Both maps are integrated by shared markers except for linkage group B10. In McClean (2007) map, only molecular markers used in association study and shared markers in blue were shown. The markers in red were found significantly (*P *≤ 0.05) associated with CBB resistance. In Freyre (1998) map, loci placed on the left side of each chromosome were shared markers in blue and molecular markers closest to previous identified CBB-QTLs. To the right of each linkage group are previously identified CBB-QTLs in different populations [3]. Symbols in subscript represent the source population of the QTL: BA Belneb-RR-1/A55, BJ BAT93/JaloEEP558, BH BAC6/HT7719, DX DOR364/XAN176, H95 HR67/OAC95, PX PC50/XAN159, S95 Seaforth/OAC95 and XC XR-235-1-1/Calima. Marker UBC420, SU91, and QTL locations are approximate because most were not directly mapped in the BAT93/JaloEEP558 population. The total distance of each linkage group is expressed in cM (Kosambi mapping function).

Since the bean lines in the CBB nursery have complex familial relationships and population structure, associations between 77 markers (75 SNP and 2 SCAR markers) and CBB rating were determined by Q + K MLM method. Very high LD was observed with 95.9% comparisons between loci significant at P < 0.01. Because CBB ratings varied between disease observation dates (Figure 1), these associations were determined for respective DAI. Tables 3 present the markers significantly associated with CBB ratings for each DAI analyses. The *P*-value determines whether a QTL is associated with the marker. The *R^2 ^*statistic is commonly used in QTL mapping studies to measure the proportion of phenotypic variation explained by molecular markers. However, unlike fixed linear regression models, linear mixed models have no well-established *R^2 ^*statistic for assessing goodness-of-fit and prediction power [29]. The *R^2^*_marker only measures the contribution of the marker to sum square after accounting for all other effects in the model [16]. Thirty-four percent (26 of 77) markers were significant in at least one date and genome-wide distributed except for LG 4. Of them, 18 and 22 markers were significantly associated with 14 and 21 DAI CBB rating, respectively. Fourteen markers were significant for both dates, especially for markers UBC420, SU91, g321, g471, and g796 (p ≤ 0.001). This suggests that CBB resistance has a complex inheritance with involvement of multiple significant loci distributed across all 11 chromosomes (Figure 4). Expression of these QTL is influenced by environment, disease pressure, plant maturity and plant organs i.e., leaves, pods, and seeds. The SCAR markers UBC420 and SU91, are known to be linked with two major QTL on B6 and B8, respectively [3], and being used for MAS for CBB resistance and to validate the presence of the QTL in resistant lines selected by phenotypic selection. The results from Q + K MLM to detect association between the marker loci and the phenotype were consistent with previously identified association of the marker loci UBC420 and SU91. Meanwhile, g471 on LG (Linkage Group) 6 and g796 on LG 8 corroborate that bean chromosome 8 and the distal region of the chromosome 6 are carrying major CBB resistance QTL [11]. Furthermore, 12 significant SNP markers were co-localized with or close to previously identified CBB-QTLs [3], i.e. g934 (CBB_BA_), g680(CBB_BJ_), g321(CBB_BH_), g2581(CBB_BH_), g2538(CBB_PX_), g2531(CBB_PX_), g1119(CBB_XC_), g696(CBB_PX_), g796(CBB_PX_), g1286(CBB_BJ_), g1215(CBB_XC_), and g1415(CBB_XC_) (Figure 4). These markers, if proved to be effective across genetic backgrounds under different environmental conditions may help breeders facilitate the pyramiding of the QTLs from diverse sources in order to attain higher levels of CBB resistance in newly-developed bean cultivars.

**Table 3:** Testing of association between marker loci and common bacterial blight severity using unified MLM (Mixed Linear Model) method

**Chr**.	CM	Marker^a^	14 DAI		21 DAI	
			
			*p*	*R^2^*_marker^b^	*p*	*R^2^*_marker
1	135	g934	n.s.		*	0.0061
2	39	g680	***	0.0098	*	0.0094
2	121	g321	***	0.0151	***	0.0141
2	123	g2581	*	0.0065	n.s.	
3	14	g1296	**	0.0104	*	0.0072
3	93	g1656	*	0.0076	***	0.0208
5	59	g1689	*	0.0090	**	0.0142
6	13	g1757	n.s.		**	0.0079

6		UBC420	***	0.0136	***	0.0215
6	105	g471	***	0.0227	***	0.0471
6	111	g1436	n.s.		*	0.0049
6	130	g2538	*	0.0075	*	0.0088
7	63	g2531	n.s.		***	0.0126
7	125	g290	***	0.0129	**	0.0085
8		SU91	***	0.0495	***	0.0320
8	46	g1119	**	0.0102	**	0.0136
8	64	g696	n.s.		*	0.0071
8	134	g580	n.s.		*	0.0048
8	166	g1713	**	0.0125	n.s.	
8	182	g796	***	0.0128	***	0.0148
9	112	g544	**	0.0101	*	0.0068
9	121	g1286	n.s.		*	0.0045
10	13	g2521	n.s.		*	0.0109
10	59	g2600	**	0.0068	n.s.	
11	61	g1215	*	0.0049	n.s.	
11	63	g1415	**	0.0073	***	0.0171

^a ^n.s., not statistically significant; *, *P *≤ 0.05; **, *P *≤ 0.01; ***, *P *≤ 0.001.

^b ^*R^2^*_marker was calculated as the proportion of sum square due to marker after accounting for all other effects in model.

## Discussion

In theory, both association mapping and linkage mapping depend on the LD between phenotypic causative and linked molecular variants [12]. Traditional mapping procedures are based on the observable differential decay of LD between loci in experimental families over one or a few generations (e.g. F_2_, RIL), while association mappings rely on historical differential decay of LD between pairs of loci in natural and domesticated populations [12]. Therefore, association mapping has the advantage over linkage mapping in that the experimental population does not need to be a set of structurally related individuals [12]. In general, association mapping is more suited for organisms with little or no pedigree information, populations with rich allelic diversity, moderate to high nucleotide diversity, and traits with little or no selection history and controlled by many loci with small effects, and lower frequencies of older alleles [12]. If there is a need to have a functional understanding of QTLs, linkage mapping is more appropriate than association mapping. This requires positional cloning of the QTL and complementation tests. This is feasible in organisms with small and/or sequenced genomes, mutants with well-defined effects and efficient transformation systems [12]. Germplasm collections and breeding populations routinely developed in our breeding program were used in this study. Since no new populations were required beforehand, association mapping makes experimental design more straightforward and saves considerable time. Moreover, the application of association mapping in QTL discovery using plant breeding populations could help integrate the process of QTL discovery with plant breeding, addressing concerns that the treatment of QTL discovery and cultivar development as separate processes may have limited the impact of MAS in plant breeding [30]. In conventional QTL mapping strategies, often, by the time a QTL mapping population is developed and mapped, breeders have introgressed the new QTL using traditional breeding and selection methods [31]. This reduced the usefulness of MAS within breeding programs at the time when MAS could be most useful (i.e., shortly after new QTL are identified) [31]. In contrast, QTL mapping strategies based on association mapping can use the populations that are routinely developed by the breeders for QTL discovery and cultivar development.

In our study, fifteen SNP markers (Figure 4) co-localized with or close to previously identified CBB-QTLs using conventional QTL mapping approaches. This suggests that association mapping using plant materials routinely developed by the breeders can effectively detect major QTLs. Moreover, since in studies of this nature, the QTL of interest is present in multiple genetic backgrounds within the breeding population, QTL detection can identify QTL that are effective across a range of backgrounds, addressing another concern with conventional QTL mapping that a significant QTL in a given mapping population may not remain effective in different genetic backgrounds. Another critical aspect for the success of association mapping is the level of LD that characterizes the species and the population used for such an analysis [12]. Considering the whole sample, we detected a very high level of LD, with most of the comparisons (95.9%) between loci significant at P < 0.01. It is even higher than a previous LD study in domesticated bean populations with 57.3% pairwise LD significant at P < 0.01 [15]. Since we worked with breeding materials, a narrower ranger of genetic diversity than previous domesticated populations were expected [15]. Although the LD is high in the bean breeding lines and generally high in the species, there will likely be regions where the LD is much reduced, such as, g1065 and g290 on LG B7, g2476 and g1656 on LG B3, and g457, g3321 and g2581 on LG2. Mutation and/or recombination may be the main mechanism that breaks down LD [12]. When LD is moderate to high, a whole genome scan can be more appropriate, whereas when the LD is low, a candidate gene approach is usually preferred, because in this case, too many markers will be needed to perform a whole genome scan to cover the variation in the entire genome [12]. Seventy-five genome-wide distributed SNPs were employed in the association study, i.e., from 6 to 8 SNPs per chromosome (Figure 4). Of the 24 previously identified CBB-QTLs [3], 62.5% (15 of 24) were confirmed by markers with significant association with them, even if they are physically distant from the QTL (Figure 4). Moreover, eight new resistance loci, g1296, g1757, g471, g1436, g580, g1713, g544, and g2521, were also identified (Figure 4). In contrast, no more than three QTLs were identified by linkage mapping in bi-parental populations [3]. Thus, due to high LD present, association study wasn't compromised by lower marker density. In addition, because bean breeding populations from several bi-parental and complex pedigrees were used in this study, association mapping has the advantage of being able to work with a higher number of polymorphic markers than conventional QTL mapping, which usually work with only one bi-parental population.

However, many of the initial associations detected have not been consistently replicated and may well have been spurious, particularly because the tests could not take sufficient account of the effect of population structural problems such as admixture [12]. In order to avoid these pitfalls, MLM method [16] was used to account for multiple levels of relatedness. K matrix was estimated from marker data. The model is able to overcome the limitations of previous association studies in plants and many other organisms, where direct calculation of co-ancestry coefficients proved impractical owing to incomplete pedigree records or inaccurate due to biases resulted from inbreeding, selection and drift [16]. Both Q and K were detected in the samples, so we fit both Q and K into the mixed model to control population structure and relatedness. Two markers, SU91 and BC420, known to be associated with CBB resistance QTLs were also included in our study. These markers were found significantly associated with CBB resistance (Table 3), which suggests that the unified mixed-model method was efficient for QTL detection. Moreover, 62.5% of the previously identified CBB-QTLs by traditional QTL analysis were also uncovered by association mapping analysis (Figure 4). This further proved that association mapping via unified mixed-model method is an efficient approach for QTL discovery in plant breeding populations.

In comparison with soybean, common bean has poorly developed genomic infrastructure (both knowledge and physical capacity). In order to accelerate association studies in bean, large-scale SNP discovery is required beforehand. Next generation sequencing is playing an increasingly significant role to speed up SNP discovery in less-characterized legumes. For instance in chickpea, Solexa 1 Gbp technology was used to sequence root cDNAs from parents of a mapping population segregating for drought tolerance [32]. One-half run of Solexa sequencing yielded 5.2 × 10^6 ^and 3.6 × 10^6 ^sequence reads for each genotype, respectively. Afterwards, about 500 SNPs were identified between parental lines [32]. In common bean, a multi-tier reduced representation library was sequenced through combining two next generation sequencing techniques, the Roche 454-FLX system and the Illumina Genome Analyzer, a total of 3,487 SNPs of which 2,795 contained sufficient flanking genomic sequence for SNP assay development [22]. Moreover, recent progress in draft genome sequencing offers important new possibilities for SNP discovery in common bean. Currently, the Joint Genome Institute is using Roche 454 technology to sequence the Andean cultivar G19833 (http://www.jgi.doe.gov/). Ultimately the availability of high-throughput and cost-effective genotyping platforms, combined with automation in phenotyping methodologies, will increase the uptake of genomic tools into breeding programs, and thus usher in an era of genomics-enabled bean breeding [32].

## Conclusions

This study demonstrated that association mapping using a reasonable number of markers, distributed across the genome and with application of plant materials that are routinely developed in a plant breeding program can detect significant QTLs for traits of interest. Unlike conventional QTL discovery strategies, in which bi-parental populations (F_2_, RIL, or DH) need to be developed, association mapping-based strategies can use existing plant breeding populations with wide coverage of the existing genetic diversity. This may address some of the concerns with conventional QTL mapping that the bi-parental mapping populations rarely give rise to new cultivars, the identified QTLs may not be effective in multiple genetic backgrounds and that the QTL-linked markers are not immediately available for MAS.

## Methods

### 1. Plant material

A population of 469 bean cultivars and breeding lines were used in this study (Table 2). These include: a) 62 navy bean varieties registered in Canada over time, since 1930, b) 29 modern North American cultivars of different gene-pool origins developed and released by public institutions in the US and Canada, and c) 378 advance bean breeding lines of different gene-pool origins, in different stages of variety development in the AAFC-University of Guelph Bean Breeding Program. These included 116 lines in the advance yield trials and 262 lines in the preliminary yield trials. The population represents the range of genetic diversity in the breeding program and the cultivars grown in Canada.

### 2. Phenotypic evaluation

A total of 395 bean lines, the advanced breeding lines in Category c, were evaluated in the field in 2009 in the common bacterial blight nursery in Harrow, Ontario in Canada. The experimental design was a randomized complete block with two replications. Each experimental unit consisted of a single 0.5 feet long row with 2 feet row-spacing. Artificial inoculation was carried out using fresh bacterial inoculum, prepared by mixing equal amount of two fuscans isolates 12 and 118, and two no-fuscans isolates 18 and 98 with spores at the concentration of 10^8 ^CFU/ml. These four strains are endemic in Ontario, Canada. Plots were mechanically inoculated at the unifoliolate growth stage using a high-pressure sprayer at the constant pressure 250 psi. Two CBB ratings were made at 14 and 21 Days After Inoculation (DAI). A 0-5 scale was used for disease severity ratings based on a visual estimate of the percentage of CBB symptoms on total leaf area, where 0 = no symptoms, 1 = less than 10%, 2 = 11-30%, 3 = 31-50%, 4 = 51-80%, and 5 = more than 80% of inoculated areas showing symptoms. CBB resistant (HR45) and susceptible (Dresden) checks were included in each block. Excel Macros programmed by QI Macros (http://www.qimacros.com/) was used to conduct Kolmogorov-Smirnov test of normality.

### 3. Genotyping

Young leaf samples (100 mg) were frozen in liquid nitrogen and ground using an AutoGrinder 48 (AutoGen Inc., Holliston, MA, USA). After incubation with plant lysis buffer (AutoGen AG00121) at 65°C for 30 min, DNA was automatically extracted using an AutoGen 850 alpha DNA automatic system following the manufacturer's manual (AutoGen Inc.).

According to McClean (NDSU) 2007 genetic map at Legume Information System (http://www.comparative-legumes.org/index.php/Home) [33], original sequence files from BAT93 and Jalo EEP558 to develop respective CAPs or dCAPs markers were re-downloaded from NCBI database (http://www.ncbi.nlm.nih.gov/) and uploaded into AlignX module of Vector NTI Advance 11 (Invitrogen, USA) for sequence alignment. Only one SNP per alignment was chosen and the preference was given to the SNP found in central region of the alignment.

Genotyping was performed using the Sequenom iPLEX Gold Assay (Sequenom, Cambridge, MA) in Genome Quebec (Montreal, Quebec). Locus-specific PCR primers and allele-specific detection primers were designed using MassARRAY Assay Design 3.1 software. DNA was amplified in a multiplex PCR and labelled using a locus-specific single base extension reaction. The products were desalted and transferred to a 96-element SpectroCHIP array. Allele detection was performed using Matrix-Assisted Laser Desorption/Ionization Time-of-Flight Mass Spectrometry (compact MALDI-TOF MS). Mass spectrograms and clusters were analyzed by the TYPER 3.4 software package that was described in details by Ehrich et al. [23]. All DNA samples were deposited on seven 96-well plates for the assay. Two lines, BAT93 and Jalo EEP558, were repeated 14 times in different 96-well plates as controls.

Two previously-characterized SCAR markers SU91 and BC420, known to be associated with CBB resistance [11] were included in the assays to provide positive controls for testing the efficacy of the analysis techniques used in this study. PCR was performed in 25 μl containing 1 μl genomic DNA (25 ng/μl), 0.5 μl dNTP mixtures (10 mM), 5 μl 5 × Green GoTaq PCR buffer (Promega, USA), 2 μl primers (1.5 mM), 0.2 μl GoTaq polymerase (5units/μl) (Promega, USA), and 16.5 μl double-distilled water. The amplification conditions were 2 min at 94°C, followed by 35 cycles of 30 s at 94°C, 45 s at 47°C, 1 min at 72°C, then 5 min at 72°C. The PCR products were analysed on 1.5% agarose gel and visualised by SYBR^® ^Safe staining (Invitrogen, USA).

### 4. Statistical analysis

Association mapping analyses were carried out with TASSEL 2.1 software, available at http://www.maizegenetics.net/index.php?option=com_content&task=view&id=89&Itemid=119. The MLM analyses were performed using a kinship K matrix and population structure Q matrix. The K matrix was generated based on 75 SNPs using kinship matrix function in TASSEL. Population structure consisted of a Q matrix that describes the percent subpopulation parentage for each line in the analysis. These percentages were calculated by STRUCTURE 2.3.3 software, available at http://pritch.bsd.uchicago.edu/structure.html. We set k (the number of subpopulations) from 1 to 10 and performed 10 runs for each k value. For each run, a burn in of 5,000 iterations was followed by an additional 5,000 iterations. Since the likelihood for model parameter k = 2 was much higher than k = 1 and comparable with k = 3 or higher, we chose k = 2 and generated a Q matrix from 75 SNPs.

The mapping information of SNP markers was extracted from McClean (NDSU) 2007 genetic map at Legume Information System (http://www.comparative-legumes.org/index.php/Home) [33]. The distribution of molecular markers, co-localized with previously identified QTLs associated to CBB resistance, was drawn by MapChart 2.1 software (http://www.biometris.wur.nl/uk/Software/MapChart/).

## Authors' contributions

CS conceived of the study, designed and carried out the lab experiments and data analysis, and drafted the manuscript. AN conceived of the study, designed and carried out the field experiments, and helped to draft the manuscript. KY conceived of the study, coordinated the study, and helped to draft the manuscript. All authors read and approved the final manuscript.

## Supplementary Material

Additional file 1 Loci, LG (Linkage Group), MAF (Minor Allelic Frequency), SNP alleles, PCR primers, and Sequenom probe sequences of 75 selected SNPs used for association mapping Click here for file
