# Supplementary material for: Association mapping of common bacterial blight resistance QTL in Ontario bean breeding populations
Source: BMC Plant Biol. 2011 Mar 24;11:52. doi: 10.1186/1471-2229-11-52 (PMC3078875; doi:10.1186/1471-2229-11-52)
Supplement: Additional file 1 — Loci, LG (Linkage Group), MAF (Minor Allelic Frequency), SNP alleles, PCR primers, and Sequenom probe sequences of 75 selected SNPs used for association mapping [file 1471-2229-11-52-S1.DOC]

| **Locus** | **LG** | **cM** | **MAF** | **Allele** | **Forward PCR primer (5’— 3’)** | **Reverse PCR primer (5’— 3’)** | **Sequenom probe (5’— 3’)** |
| --- | --- | --- | --- | --- | --- | --- | --- |
| g2562 | 1 | 33 | 36.60% | T/A | ACGTTGGATGATATTTGACGCCAAGGCAGG | ACGTTGGATGTGGTGTAGGACCATTACCTG | CCTTTGTCATACCACGAAACAAGTTT |
| g1404 | 1 | 72 | 33.54% | G/C | ACGTTGGATGTGGTGTGATGAGGAGGTATG | ACGTTGGATGGTGACACTAGCATAAAACTC | TGAAAATCAAATCAGATCAAGACA |
| g1886 | 1 | 89 | 36.40% | T/C | ACGTTGGATGCCGGGAACAGTATTTTGAGG | ACGTTGGATGGATAAACCTCCGACCTCTTC | ATAGAGTTCAAGAAGCGTAATCGTTC |
| g724 | 1 | 105 | 35.40% | G/A | ACGTTGGATGACAGAGTGATCTGTGACCTG | ACGTTGGATGACCCCTTTTAGTCAATTCGC | GCTTCTTTGTTTACTTTCTATTAGTA |
| g1959 | 1 | 107 | 36.20% | G/C | ACGTTGGATGCAGTGCTAGCAATGATGCAG | ACGTTGGATGACGCAGGAAAGTTGGGTTTG | GCAGAAGCTGGATAAGA |
| g934 | 1 | 135 | 37.43% | G/C | ACGTTGGATGACCGTCCAACTAGAAACTCC | ACGTTGGATGAAGACCCTAAGCTGTTCGAG | GGATGGGCGTGTACACGGC |
| g1645 | 1 | 165 | 35.69% | C/T | ACGTTGGATGGATCCAATTCCAGAGACACC | ACGTTGGATGGAAGAAAGCTCGTAAAGCCC | GTTGGAGGTTGGGATTTCACATC |
| g1795 | 1 | 171 | 35.85% | C/G | ACGTTGGATGAGAAGGAGTATGTGGTGGAC | ACGTTGGATGATAATCTGCGTGAGCTCACC | CCAACTTCTTCCTGAACTTCTC |
| g774 | 2 | 34 | 36.69% | C/T | ACGTTGGATGAAGAAGATCGATCCGTGAGC | ACGTTGGATGATGACCAGAGGGATGAAACC | ACCGTAATGAGTGATGACCTT |
| g680 | 2 | 39 | 43.50% | C/T | ACGTTGGATGGCCACAGATCCTCAGAAATC | ACGTTGGATGGAAGAGAAACAAAAGTAGCAC | CCTTTATTTGCAGAACAATTAGTTACCT |
| g457 | 2 | 111 | 37.17% | G/A | ACGTTGGATGCCTCCCGGTTAGTTACATAC | ACGTTGGATGGTCTTGAACACTCAAACCTG | AAACGAACACTCAAACCTGGAAATGACAT |
| g321 | 2 | 121 | 34.25% | C/T | ACGTTGGATGTGGTGACTCAGCTGAGGGA | ACGTTGGATGAATCCACCACCATCTTCACC | AGCTCCTGCTCCAGCTTCTTCTCCT |
| g2581 | 2 | 123 | 35.32% | T/C | ACGTTGGATGAGTCACCTAAGCAACCTCTC | ACGTTGGATGAGAGACCTGTCCCATTGTTG | TCAGCATGCTTCAACATCTG |
| g2020 | 2 | 202 | 37.55% | C/T | ACGTTGGATGTTGATTGGAGTAAGGCACCC | ACGTTGGATGATTAGAGGCACAGTTGGCAG | CCAAGTTGGCAGGTCCTGG |
| g1296 | 3 | 14 | 46.97% | A/G | ACGTTGGATGGGGATGAGAATGGTAAAGCC | ACGTTGGATGAATTCTTGCAAGGCACACCC | CAATTCTCCCTCCCAG |
| g1808 | 3 | 55 | 45.19% | C/T | ACGTTGGATGATGTCCTCTGCCACGTAAAC | ACGTTGGATGCCAAGAATCAACTGGCTGTG | GGACAGGGAACTTCGTGGCCT |
| g2476 | 3 | 87 | 38.85% | C/G | ACGTTGGATGCACTTTCCTTGATGCTGCTC | ACGTTGGATGGCTTGAGTTCAGTCTCTTCC | AAGCCTGAACCTCCAAGAACA |
| g1656 | 3 | 93 | 42.37% | G/A | ACGTTGGATGACCACTTCCCATGTGAAGTC | ACGTTGGATGCAACCTTGATCTGAAGAGGG | CGAAGAGGGGTGGGAAT |
| g586 | 3 | 104 | 34.90% | T/C | ACGTTGGATGGGAAAAATCATGCACCTATC | ACGTTGGATGACCAGGTATATAACCGCATC | TCTATAACCGCATCCACCTA |
| g2108 | 3 | 138 | 32.78% | C/T | ACGTTGGATGAAATGTTCACGCCGAAGAGC | ACGTTGGATGTGGAAGGCGCGGAATAATAG | GAACGGTGGTTGAACCGAT |
| g2274 | 3 | 146 | 31.51% | C/T | ACGTTGGATGCCCCACATGTTTGTGAATGC | ACGTTGGATGCCTTCAGATACTCCTTGACC | GCGCAACAGAAAGAAGATT |
| g968 | 4 | 0 | 34.86% | A/C | ACGTTGGATGGAATTCGTGCATGCTAAACC | ACGTTGGATGTCTGCAACTTCCACTCTCTC | GCTGGTACATTCTGCGACACTAAAA |
| g755 | 4 | 22 | 21.67% | G/A | ACGTTGGATGGGAAAGCGGGTTAGGTATAG | ACGTTGGATGATATGCTCACAGTAGCATGG | CCCCATATGATTATTAAACACACAATAAGA |
| g2595 | 4 | 53 | 35.42% | C/G | ACGTTGGATGTGGAGCATGCTAGCCTTTTG | ACGTTGGATGGGCATTACACACTCAAACAC | TATAACAAATCACCACCTTAACT |
| g128 | 4 | 74 | 41.11% | G/C | ACGTTGGATGCCCCCTTCTCCATATAGTTC | ACGTTGGATGGGAAGACTTCAAATATGCTC | CTTAAAATATGCTCATCAACAGGTTTGT |
| g483 | 4 | 90 | 31.63% | G/A | ACGTTGGATGACCCAAATTCGCAGAAATCC | ACGTTGGATGCGGGTTTGAGAAGTTTAGGG | GCACCATTTCTACAACTGGTATTGA |
| g1375 | 4 | 103 | 30.63% | G/C | ACGTTGGATGTGAACCACTCCGATGCAATC | ACGTTGGATGCTACAAGAAGCCTTGGAGAG | GTGCTTGTGGCTCATCGTTTGTC |
| g2467 | 4 | 117 | 49.12% | T/C | ACGTTGGATGGATGCAGGCCAAAGTTAAGG | ACGTTGGATGTGAGAGATGGCTTGGTGAAC | TGGCTTGGTGAACAAAACA |
| g1188 | 5 | 16 | 35.04% | A/T | ACGTTGGATGCTCCATGTTGGTCTATCTCC | ACGTTGGATGGATTGTGTGAGAGCAGAACC | ACCGAACCTGCGATCTACA |
| g1968 | 5 | 45 | 34.10% | G/T | ACGTTGGATGTAGTGCTAACTCTTGCTAGG | ACGTTGGATGGGGCTGCGAAGTGAAAAAAG | TGAAGAAATAGAGTATTCGGAT |
| g1333 | 5 | 52 | 25.68% | C/T | ACGTTGGATGGATGTGAAAGTGGAATAGGC | ACGTTGGATGGCTTTCATGTCTGCAAGGTC | GAGGATACATTTTCTTTTATTATTGCTA |
| g1689 | 5 | 59 | 40.41% | G/C | ACGTTGGATGTGCTGTGATGTGTCATGGTC | ACGTTGGATGAGAAGCAGAAAAACACGTGG | GAAGAAGCAGAAAAACACGTGGTGGCCCCG |
| g1664 | 5 | 66 | 31.96% | C/G | ACGTTGGATGACACGTCTCAGGTTCCTAAG | ACGTTGGATGTGGGATAACGAACACTCAGC | AACCTTACATCATCTATCACAGGTCCACA |
| g1883 | 5 | 77 | 33.66% | T/A | ACGTTGGATGGTGGCAGGTAGTCAACTTTG | ACGTTGGATGGGTTTCCAGCGAAGGAATTG | GGTCACTGAGCTACCAGG |
| g1757 | 6 | 13 | 46.44% | A/G | ACGTTGGATGTCCACAATGGCTCAATCTCC | ACGTTGGATGAGATGGAGCCGGAGACAATG | GGAAGGAGACAATGACCTCCTCGG |
| g2208 | 6 | 29 | 35.89% | C/T | ACGTTGGATGGCAAAAATCATGCAGCAGCC | ACGTTGGATGAGGTGCAACTGCATCACAAC | CTCTAATGGCTCCGCAGA |
| g1998 | 6 | 37 | 36.50% | C/T | ACGTTGGATGTGCCCACTGAAAAGATCGCC | ACGTTGGATGTACCCAACACAGAGACTAAC | AAAGCGAGATCAATTTCCAC |
| g471 | 6 | 105 | 41.68% | G/A | ACGTTGGATGTGAGGAAACTAGAGGTGTGC | ACGTTGGATGTGCAGTTACAGTCTTCCTCC | CCGTAATTCACTGCATCTCGAATCAC |
| g1436 | 6 | 111 | 39.92% | G/A | ACGTTGGATGGGGTTGCAAGGTTTCACTTA | ACGTTGGATGAATCAGAGCCATCACAACCC | CCATCACAACCCTGTATAC |
| g2538 | 6 | 130 | 30.92% | A/G | ACGTTGGATGACGAGGAGGTTGATGAGATG | ACGTTGGATGTTACTCCTCACTTGGCCATC | CATCGTTGATCTGCCCATCGCCATC |
| g503 | 7 | 0 | 32.39% | G/C | ACGTTGGATGTTGCCAACTGGAAGATCTCG | ACGTTGGATGATGCTCAGCTGCAGAGCTTC | CCCAGAACTTGAGCCT |
| g134 | 7 | 10 | 32.39% | G/C | ACGTTGGATGTTGCCAACTGGAAGATCTCG | ACGTTGGATGATTCTCAGCTGCAGAGCTTC | GGGAGCCCAGAACTTGAGCCT |
| g1615 | 7 | 31 | 29.45% | A/C | ACGTTGGATGACATGGCGGTGCTTTACTTG | ACGTTGGATGTTCCTCTTGCTGTCCTTCAC | TGCGTCTTTCCTCCGGA |
| g2129 | 7 | 38 | 40.78% | G/A | ACGTTGGATGGGTGCTCCAAGAATGGTATG | ACGTTGGATGCACTCAATCCAAACCAAGCC | CCAAGCCCTAATCCACA |
| g2531 | 7 | 63 | 41.83% | A/C | ACGTTGGATGTCTTTCTCGGTCCTGAATCC | ACGTTGGATGAGGTGAGTGTAGTGTCTTTG | CTGCAACTCTAACCCTG |
| g1065 | 7 | 122 | 30.87% | A/C | ACGTTGGATGGCTGAGTCAACAAGTGCAAC | ACGTTGGATGTTCTGGCAAACAACTACCCG | CCCTGTCGTGTGACTTTATAACTCCCAATG |
| g290 | 7 | 125 | 34.12% | T/C | ACGTTGGATGGGACGTGAAAGATCACATTG | ACGTTGGATGGGTGGTGCACACAATTATCC | AAGATAAGAAAGCTTAGGCT |
| g2311 | 8 | 28 | 39.22% | T/G | ACGTTGGATGCTGTGACATGACAACTTCGG | ACGTTGGATGGTCTTACAACTTCAGCCTGC | CCTTCAGAAACTGTAACAACATTGCTTCA |
| g2393 | 8 | 34 | 37.35% | G/C | ACGTTGGATGAGGGTGATGTGGACACAATG | ACGTTGGATGGCATGTAAGGTGTTCATGGG | AGTCATGGGGCCCTGCTTGGTCTCTAGTAA |
| g1119 | 8 | 46 | 33.14% | C/A | ACGTTGGATGGGTCCATGTTGAGTGAAAGC | ACGTTGGATGCCGAGAAGAACCATTCTGAG | AGCATTGATGAGTTTTGGAACA |
| g696 | 8 | 64 | 40.49% | C/T | ACGTTGGATGTCTTTTTGCTTCCGCGGATG | ACGTTGGATGCTAAGATCCCCTTCGAGGAG | AGGTCGCGAACCTTCA |
| g580 | 8 | 134 | 36.01% | T/C | ACGTTGGATGCAACACAGTCTCGTAAACCC | ACGTTGGATGCGTATGCAGGAAAAGTACGG | AGCTCGTTCTCGCTAAATCC |
| g1713 | 8 | 166 | 43.47% | C/T | ACGTTGGATGACAGGGCAAAACTGGATGAC | ACGTTGGATGTAAGTGCCAAGTCCTTGGTC | CTTGGTCATCTTCCCAGC |
| g796 | 8 | 182 | 36.18% | A/G | ACGTTGGATGCAGAACGGTCTTAACTACGC | ACGTTGGATGCCTCCAAAGTGTTGGGATTG | CGTCTGGTTGAATGGC |
| g195 | 9 | 16 | 36.20% | C/T | ACGTTGGATGTGAGAAGGTGTCAACTTTCG | ACGTTGGATGCTCTTGGACAGTACCACTAC | GGCGTTAGTTTCTGCAGTTCCATC |
| g1379 | 9 | 28 | 36.96% | C/T | ACGTTGGATGTTGCTGGTGACCTTGGCAAC | ACGTTGGATGAAATGAGCAACGCACAGACG | CCAGCGCCGCCCTCACTGA |
| g1206 | 9 | 60 | 36.30% | C/T | ACGTTGGATGGCAGTTCGGTTACTTCAAGC | ACGTTGGATGTCTTCTTCTCGGCGATCTTG | TGGCCTTGACGACGAGGGG |
| g792 | 9 | 70 | 34.18% | G/T | ACGTTGGATGTGACATTGGTTGATCCCCTG | ACGTTGGATGTTTCTCTGAGTCTGTCTGCC | CCCCCTCACCGAACTTGCAA |
| g2498 | 9 | 87 | 35.19% | T/C | ACGTTGGATGAAACTCTGATCCCGTAGCAC | ACGTTGGATGTGCCACAAGCAGAATTACCC | TCCCTTATGCTGTTTTTTACTG |
| g544 | 9 | 112 | 36.69% | C/G | ACGTTGGATGGATAACAGATCCACCACTGC | ACGTTGGATGGGGACCTTTTTGCAAGTGG | GTTGTATGGAGTGATCAAGGGTCT |
| g1286 | 9 | 121 | 45.20% | T/A | ACGTTGGATGAACTGAGTGGCACTGGATAC | ACGTTGGATGTGATAATACCCGGTTGGAGG | GACTGTTGGAGGTCCCATTCCATTCAC |
| g2521 | 10 | 13 | 30.94% | T/A | ACGTTGGATGGGTGTTGGTAATCATGTGCC | ACGTTGGATGAGCTAGAGTTCATGCTTGTG | CCTCGTGTTTTTTTGCTTAGACATC |
| g1320 | 10 | 21 | 44.72% | T/C | ACGTTGGATGTGTTGTCAGTGGCATTGGTG | ACGTTGGATGTGTCCTTCCCATGAACTTCC | CAATCCACTGGTAACTGCT |
| g1029 | 10 | 29 | 35.84% | T/A | ACGTTGGATGAAAGCATGGGGTTACAACTG | ACGTTGGATGGCGCCTCTGCAAATTGTATG | CCCTAGCGTGGGAAAGGTAGTTTTTAAC |
| g1994 | 10 | 42 | 38.76% | G/C | ACGTTGGATGATCAGAAGCAGCCAAGTGAC | ACGTTGGATGCAGATCACACTAGACCTACC | ACCTACTATCAACAGTCATATATAT |
| g2600 | 10 | 59 | 30.68% | C/T | ACGTTGGATGGGAAGGAACAACAATTCAAG | ACGTTGGATGCTCTGAAAGTGAGACCTTCC | CCCCCAGGGCATCTTAACTGG |
| g2560 | 10 | 64 | 24.07% | C/A | ACGTTGGATGTGGGAGAAGTTCATTGCCAG | ACGTTGGATGAGTATATGGCTGGATCCCTG | ATCCCGGCAACCAAAAACCACTT |
| g1724 | 10 | 72 | 29.29% | C/T | ACGTTGGATGAGAAACTCCCTCGCAATGCC | ACGTTGGATGCTAGAAGATATGTCAAGGTG | AGGCCAAGATCGAAGCAAAAGTAAA |
| g2260 | 10 | 84 | 35.42% | T/A | ACGTTGGATGATGGCATCAAAAGGAGAAGG | ACGTTGGATGGTGGCATCTTTGCTTATGCG | GGAGGAATGATTCCCTCTCGTTGAGGTAT |
| g2273 | 11 | 48 | 33.27% | C/T | ACGTTGGATGTCACGATTCACGACTGCTTC | ACGTTGGATGCACCAATAACCAATTGCAGG | TAATTATATTAGACAAACTAGCATCT |
| g1215 | 11 | 61 | 36.79% | C/G | ACGTTGGATGTTCACGACGGGATTCTCCCT | ACGTTGGATGACCTTTTCTGCGCTGAGCAC | GTCTGCGCTGAGCACCGGTACAC |
| g1415 | 11 | 63 | 37.96% | T/A | ACGTTGGATGACAAGCAAACTGATCAGTCG | ACGTTGGATGACGCAGGAACCTTTTTAGTG | AGCTGATCAAGCTTTCC |
| g1438 | 11 | 74 | 38.14% | C/T | ACGTTGGATGATGTCCCCATTCCCATATCC | ACGTTGGATGCTTCCACAAACCCAGGTTTC | TCCTTGGAACCCAAAG |
| g1168 | 11 | 78 | 30.53% | C/T | ACGTTGGATGTAATCCATAGGAGGAGCCAG | ACGTTGGATGCAGAGTGACTCAGATTCCTC | CTCCGTTGTTGATTGCCTA |
| g156 | 11 | 82 | 28.29% | T/C | ACGTTGGATGCCTTTTGAGGAGTCCTTGTG | ACGTTGGATGTTCCAGCTCCAGTAAACACC | AGCCTATACAAGGTTAAAACAGTT |
